# Closing the Fibre Gap—The Impact of Combination of Soluble and Insoluble Dietary Fibre on Bread Quality and Health Benefits

**DOI:** 10.3390/foods13131980

**Published:** 2024-06-24

**Authors:** Rebecca Sempio, Celia Segura Godoy, Laura Nyhan, Aylin W. Sahin, Emanuele Zannini, Jens Walter, Elke K. Arendt

**Affiliations:** 1 School of Food and Nutritional Sciences, University College Cork, College Road, T12 K8AF Cork, Ireland; rebeccasempio@umail.ucc.ie (R.S.); cseguragodoy@ucc.ie (C.S.G.); lnyhan@ucc.ie (L.N.); asahin@ucc.ie (A.W.S.); e.zannini@ucc.ie (E.Z.); 2 Department of Environmental Biology, Sapienza University of Rome, Piazzale Aldo Moro 5, 00185 Rome, Italy; 3 School of Microbiology, Department of Medicine, University College Cork, T12 Y337 Cork, Ireland; jenswalter@ucc.ie; 4 APC Microbiome Ireland, University College Cork, T12YT20 Cork, Ireland

**Keywords:** bread, high fibre, response surface methodology, fortification, shelf life, reducing sugar release

## Abstract

Dietary fibre (DF) is important for overall health and disease prevention. However, the intake of DF in Westernised countries is below the recommended level, largely due to the excessive consumption of low-fibre foods. Fortifying staple foods, such as bread, with dietary fibre ingredients is one approach to closing the fibre gap in our diet. However, incorporating purified and chemically modified fibre ingredients into food is challenging. This study unveils interactions between soluble–fermentable (arabinoxylan), insoluble–fermentable (resistant starch type IV) and insoluble–unfermentable (cellulose) fibre ingredients and their impact on bread quality using Response Surface Methodology. This resulted in an optimised mixture of these fibre ingredients that can coexist within a bread matrix while maintaining quality characteristics comparable to white wheat bread. The partial replacement of flour with fibre ingredients led to an interference with the gluten network causing a reduction in gluten strength by 12.4% and prolonged gluten network development time by 24.4% compared to the control (no fibre addition). However, the CO_2_ retention coefficient during dough fermentation was not affected by fibre ingredient inclusion. The fibre content of the white bread was increased by 128%, with only a marginal negative impact on bread quality. Additionally, the fibre-fortified bread showed a lower release of reducing sugars during in vitro starch digestion. This study illustrates the synergy of different types of fibre ingredients in a bread system to advance in closing the fibre gap.

## 1. Introduction

Dietary fibre (DF) is widely acknowledged as a fundamental component of a healthy and balanced diet, with recommended intake levels established by nutritional guidelines worldwide [1,2]. However, the disparity between suggested fibre intake and actual consumption is a concern [3]. Evidence illustrates that in Europe, only between 9% and 13% of the adult population meets the recommended amount of fibre [4,5,6,7]. This fibre gap has driven initiatives like the “Action on Fibre” for food reformulation [8], emphasising the importance of closing this nutritional divide.

Although high-fibre products are promoted for their health benefits, consumer compliance is often hindered by off-taste and texture issues, such as bitter taste and dry texture [9,10,11]. Therefore, incorporating fibre into products is challenging as it can compromise the products’ structural integrity, thus compromising their market appeal. One way to help people increase their intake of DF is to consider fortifying cereal-based products with refined and modified fibre ingredients [12]. The substitution of refined flour with fibre ingredients can lead to a reduction in available carbohydrates and a superior nutritional value of cereal-based staple foods.

However, as reported by Sempio et al. (2023) [13], current research primarily focuses on the application of single fibre ingredients rather than a combination of them. This tendency may arise from the fundamental understanding of the impact of individual fibre ingredients on food properties. Equally, studies on individual fibres can serve to characterise specific outcomes related to a single fibre ingredient.

The soluble fibre ingredient that seems to be investigated the most is arabinoxylan. Arabinoxylan supplementation has been associated with health benefits such as improved glucose and insulin metabolism [14,15]. It is recommended that consumers reach an intake of 8 g of arabinoxylan per day to secure its health benefits [16]. Evidence illustrates that arabinoxylan addition to bread influences other polymers such as starch and gluten [17,18,19,20]. Specifically, a small amount enhances physicochemical properties, but excessive doses can reduce specific volume and affect the crumb structure by increasing the number of holes [21,22]. Insoluble fibre, such as cellulose and resistant starch, is also widely studied in the bread matrix. Cellulose has been shown to slow gastric transit time, promoting satiety and facilitating a gradual release of glucose [23,24,25]. Additionally, its water-retaining properties augment stool volume, thereby contributing to the mitigation of constipation and risks associated with colonic cancer and diverticulitis [26]. However, even in small amounts, cellulose significantly affects the dough structure, resulting in reduced volume and increased hardness [27,28,29]. Resistant starch has been proven to reduce post-prandial glycaemic responses [30]. Consequently, the European Food Safety Authority has established a claim that “high carbohydrate baked foods should contain at least 14% of total starch as resistant starch in replacement of digestible starch” [31]. An addition of up to 20% can be used without significantly impacting the bread characteristics [32,33,34,35].

However, the application of single fibre ingredients restricts the potential benefits that a combination of fibre ingredients can offer, encompassing both nutritional and technological characteristics. Research on fibre combinations can reveal synergistic effects and comprehensive health and techno-functional improvements, paving the way for innovative functional foods and more effective dietary interventions.

This study examines the effects of individual fibre ingredients (soluble AgriFiber Soluble Fiber Corn (SFC) (AgriFiber Solutions, Mundelein, IL, USA), insoluble Fibersym^®^ RW (MGP, Atchison, KS, USA) and VITACEL L 600-30 (J. Rettenmaier & Söhne GmbH + Co KG, Rosenberg, Germany)) and their combinations within a bread system. Response Surface Methodology (RSM), a statistical tool, was used for experimental design and randomisation of different inclusion levels of the fibre ingredients in combination runs. This software compares the relationship between individual variables (AgriFiber SFC, Fibersym^®^ RW and VITACEL L 600-30) and the response reported in the following section (bake loss, specific volume, bread crumb structure, bread crumb hardness and water activity). The RSM contribution in this research provided the fundamental understanding of fibre inclusion on the bread quality, which led to the optimisation of a fibre mixture mimicking a white wheat bread. This represents the advances in fibre research in a bread matrix. The impacts of the DF ingredients on dough properties and bread characteristics were assessed, including nutritional composition, texture and colour, as well as their impact on in vitro digestibility and shelf life.

## 2. Materials and Methods

### 2.1. Raw Materials

The ingredients used for the preparation of the bread were baker’s flour (BF) (Odlums Group, Dublin, Ireland), water-soluble arabinoxylan from corn known as AgriFiber Soluble Fiber Corn (SFC) (AgriFiber Solutions, Mundelein, IL, USA), resistant starch type IV known as Fibersym^®^ RW (MGP, Atchison, KS, USA) and cellulose known as VITACEL L 600-30 (J. Rettenmaier & Söhne GmbH + Co KG, Rosenberg, Germany). Vital gluten (Roquette, Lestrem, France) was added to the different fibre-containing formulations to compensate for the reduction in protein content from substituting the baker’s flour. In addition, all bread formulations included instant active dried baker’s yeast Saccharomyces cerevisiae (Puratos, Groot-Bijgaarden, Belgium), salt (Glacia British Salt Limited, Middlewich, UK), sugar (Siúcra, Dublin, Ireland), sunflower oil (Musgraves, Cork, Ireland) and tap water. The protein concentration of BF, DF ingredients and vital gluten (data reported in Table 1) was quantified following the Kjeldahl method [36]. This method was performed following three steps: digestion, neutralisation and titration. Digestion of the sample with sulphuric acid was performed to convert nitrogen into ammonium sulphate, neutralisation and distillation to release ammonia, and titration to measure the amount of ammonia, thereby quantifying the nitrogen content. The fibre concentration of these ingredients was quantified by AOAC method 2022.01 with minor amendments, as reported by Sahin et al. (2023) (data reported in Table 1) [37,38]. All the experimental chemicals were purchased from Sigma Aldrich (St. Louis, MO, USA) unless otherwise stated.

### 2.2. Experimental Design

The experimental design of fibre fortification in a bread formulation was performed using the software Design Expert 9 (StatEase, Minneapolis, MN, USA). A three-factorial, face-centred, central composite design was chosen with single factorial points and four repetitions of the centre point. Concentrations of AgriFiber SFC (0–5%), Fibersym^®^ RW (0–40%) and VITACEL L 600-30 (0–20%) were used as variable parameters of the experiment. All DF ingredient concentrations are reported based on flour replacement. The maximum concentration of each fibre type was selected based on preliminary experiments, ensuring that concentrations remained below the threshold where bread quality was significantly compromised. Table 2 displays the different bread formulations across the 18 different factorial points. Prior to bread production, the water content of all runs was adjusted using a Farinograph-TS^®^ as detailed below. The impact of the fibre ingredients in different combinations on bread quality was evaluated by determining bake loss, specific volume, bread crumb structure, bread crumb hardness and water activity, as described in the following sections. To determine the level of coexistence of all three fibres, Response Surface Methodology was applied. Therefore, models were produced applying backward elimination regression of insignificant model terms with an α to exit of 0.1. For significant models with an insignificant lack of fit (LOF), 3D response surface plots were produced.

### 2.3. Adjustment of Water Content

A Farinograph-TS^®^ (Brabender GmbH and Co KG, Duisburg, Germany) equipped with an automatic water dosing system (Aqua inject) and an S300 mixing chamber was used to determine the water absorption of the various formulations. All measurements were conducted using a total of 300 g dry ingredient mixture (flour, vital gluten and fibre ingredients) standardised to 14% moisture content. For each test, the dry ingredients were pre-blended for one minute in the chamber to ensure homogeneity before water injection. The water addition process was precisely controlled by the Aqua inject system, which aimed to achieve a target torque of 500 ± 20 FU at which the optimal water concentration was reached. The kneading chamber was maintained at a constant temperature (30 °C). This target torque was selected based on the literature target to ensure optimal dough consistency and moulding properties [39]. Firstly, a titration was performed to estimate the water addition percentage, followed by two runs with the addition of total water percentage. All formulations of the experimental design including the 100% baker’s flour control bread (BFB) and the optimised fibre-enriched bread (FeB) mixture are reported in Table 2. To compensate for the protein loss caused by the replacement of flour by pure fibre ingredients, vital gluten was added, aiming for the protein content of the control (baker’s flour).

### 2.4. Dough Preparation and Bread Production

Bread dough was prepared using a Kenwood Chef Xl 67L 1200W Food Mixer (Kenwood Ltd., New Hampshire, UK). The control recipe (BFB) is reported in Table 3. First, dry ingredients were mixed at speed 1 for 1 min with the whisk attachment. Instant dried yeast was hydrated and activated in water (25 °C) for 10 min. Subsequently, the yeast solution and sunflower oil were added to the dry ingredients. The ingredients were mixed with a hook attachment, initially at speed 1 for 1 min, followed by a second mixing stage at speed 2 for 7 min. The same procedure was followed for the fibre-enriched runs, where a blend of flour, vital gluten and fibre ingredients was used according to the experimental design illustrated in Table 2.

A dough volume of 1 kg was divided into two portions of 450 ± 1 g each. These portions were then moulded, transferred to greased tins, and subjected to a 90 min proofing process in a proofing chamber (KOMA SunRiser, Roermond, The Netherlands) maintained at 35 °C and 75% humidity. Following proofing, the bread loaves were baked in a deck oven (MIWE Condo, Arnstein, Germany) at a temperature of 220 °C/230 °C (top/bottom) for 35 min. An injection of 400 mL of steam was carried out preceding the loaf loading into the oven, and the oven draft remained open throughout the baking process. After baking, the bread loaves were removed from the tins and cooled for 1 h before analysis. Formulations displayed in Table 2 indicated as ‘runs’ were baked once (except for the centre point, which was baked in quadruplicate).

### 2.5. Impact of Different Fibre Combinations on Bread Quality

#### 2.5.1. Bake Loss

The bake loss was examined to investigate the amount of water lost during the baking process. The bake loss of two loaves per batch was calculated according to the following formulas:

Weight of the dough(g)−Weight of baked bread(g)=Moisture lost during baking(g)

$$\frac{\mathrm{Moisture}\mathrm{loss}\mathrm{during}\mathrm{bake}\;(g)}{\mathrm{Weight}\mathrm{of}\mathrm{dough}\;(g)}\times 100=\mathrm{Bake loss}\;\left(\%\right)$$

#### 2.5.2. Specific Volume

The specific volume was investigated using the Volscan Profiler (Stable Micro Systems, Surrey, UK) and expressed as mL/g. Two loaves were analysed per batch.

#### 2.5.3. Crumb Structure

The slice area (mm^2^), number of cells and cell diameter (mm) of central slices were investigated using the C-Cell Imaging System (Calibre Control International Ltd., Warrington, UK). Two loaves per batch were cut into slices of 25 mm thickness, and five slices per loaf were analysed. The crust slices were omitted.

#### 2.5.4. Crumb Texture

The crumb texture of bread slices was evaluated using the TA-XT2i Texture Analyser (Stable MicroSystems, Surrey, UK). The test was performed as described by Neylon et al. (2023) [39]. Ten bread slices of 25 mm thickness per batch were analysed for their hardness and resilience. The crumb hardness was assessed as the maximum force during the initial compression. Bread crumb resilience was calculated by dividing the energy needed during the upward movement after the first compression by the energy needed during the compression.

#### 2.5.5. Water Activity

Water activity (a_*w*_) was evaluated using AquaLab series 3 (Decagon Devices Inc., Pullman, WA, USA). For each loaf of bread, 2 g of crumbs was placed in a closed container and allowed to rest for 5 min for equilibration prior to the test run.

### 2.6. Combination of Fibre Ingredients

The optimal blend of fibre ingredients and the addition level which minimally impacts bread quality were determined using the computer simulation software Design Expert v. 9. The aim was to replicate the quality of the control bread (BFB) with the maximum possible addition of DF. Therefore, maximising specific volume and number of cells, and decreasing crumb hardness were selected as the desirable factors. Due to significant changes in colour with the addition of AgriFiber SFC, its inclusion was restricted to 2%. As a result of the RSM optimisation tool, flour was replaced by 2% AgriFiber SFC, 40% Fibersym^®^ RW, and 11% VITACEL L 600-30, resulting in the fibre-enriched bread (FeB) formulation. In addition to the bread analyses previously listed, the following dough analyses and additional bread analyses were performed on BFB and FeB. The control bread (BFB) and the bread fortified with the final combination (FeB) were baked in triplicate.

#### 2.6.1. Interaction of Fibre Ingredients with Protein and Starch

##### Interference with Gluten Network Development

To determine the strength and development time of the gluten network of BFB and FeB, GlutoPeak (Brabender GmbH and Co KG, Duisburg, Germany) was used. Flour, fibre ingredients and vital gluten were weighed out with a final volume of 9 g (based on 14% moisture) and premixed to ensure homogeneity before analysis. Deionised water (36 °C) was added to bring the total volume to 18 g, and the test was started using a shear speed of 2750 rpm at 36 °C. The torque was monitored over time (s), and torque maximum (TM) in Brabender Units (BU) and peak maximum time (PMT) in seconds (s) were evaluated.

##### Impact on Dough Development and Starch Pasting Properties

The dough mixing, pasting behaviour and baking quality of BFB and FeB were analysed using Mixolab (Chopin, Villeneuve-la-Garenne CEDEX, France), following the method outlined by Rosell, Santos and Collar (2010) [40]. The standard protocol “Chopine+” was selected. Briefly, flour, fibre ingredients and vital gluten with the adjusted water content (Table 4) were prepared to reach a total dough volume of 75 g. Samples were mixed at a constant speed of 80 rpm while a heating profile was applied. The starting temperature was set to 30 °C and maintained for 8 min, after which the temperature was raised at a rate of 4 °C per minute until it reached 90 °C. The temperature was maintained at 90 °C for a duration of 7 min, followed by a cooling step to 50 °C with a cooling rate of 4 °C/min. The final temperature of 50 °C was held for 5 min. As described by Rosell, Collar and Haros (2007), the following parameters were evaluated: the time (min) required for the maximum torque to be achieved during the initial mixing stage at 30 °C known as dough development time (DDT), the minimum torque during the mixing and heating phase (C2), maximum torque during the mixing and heating phase at 90 °C (C3), minimum torque reached in the cooling to 50 °C (C4), and maximum torque reached in the cooling to 50 °C (C5) [41]. C2 measures protein destabilisation or weakening as a result of mechanical work and temperature. C3 provides information on starch swelling and hydration properties. C4 gives insights into the dough’s physical breakdown of starch granules. Finally, C5 offers insights into the extent of starch retrogradation.

##### Effect on Yeast Performance

The fermentation quality of the BFB and FeB bread doughs were analysed using a Rheofermentometer (Chopin, Villeneuve-la-Garenne CEDEX, France). A total of 300 g of dough was prepared and placed into the fermentation chamber, and a 1500 g cylindrical weight was positioned on top of the dough. The dough was allowed to ferment for 3 h at 30 °C. The analysis included an evaluation of the maximum dough height (Hm) (mL), the volume of CO_2_ produced during fermentation (mL), and the CO_2_ retention coefficient (%).

##### Changes in Viscoelastic Properties

The viscoelastic properties of BFB and FeB doughs were investigated using a Rheometer Physica MCR 301 (Anton Paar GmBH, Ostfildern, Germany). The dough was prepared without the addition of yeast. Before the analysis, dough samples were left to rest in a covered mixing bowl for 5 min after the mixing step. Serrated plates were used in a parallel geometry setup to avoid slippage; the temperature of the lower plate was maintained at 35 °C, accompanied by an upper plate of 50 mm in diameter. Dough samples were loaded onto plates. Before running the test, the plates were brought into position, and the sides of the samples were covered with mineral oil to avoid dehydration. Frequency sweeps were performed as described by Neylon et al. (2021) using a constant strain of 0.01% to investigate the extent of changes in viscoelastic properties of the different dough; the damping factor $\tan\delta \frac{G^{\prime \prime }}{G^{\prime }}$ was evaluated [42].

#### 2.6.2. Bread Analyses

In addition to the bread analyses performed on the breads in the experimental design, the following bread characteristics were determined.

##### Compositional Analysis

Compositional analyses of BFB and FeB were completed externally by an accredited laboratory (Chelab S.R.L., Resana, Italy). Two fresh loaves of bread for both control BFB and FeB were provided to the external laboratory and the following analysis were performed. For total protein quantification, a method based on AOAC 990.03, AOAC 992.15, and AOAC 992.23 was used [43,44,45]. The fat concentration was determined by a method reported as MP 2598 rev 0 2022. Ash quantities were assessed by a method developed based on AOAC 945.46, AOAC 923.03, AOAC 938.08, and AOAC 920.93 A [46,47,48,49]. Moisture was calculated using a method which was developed based on AOAC 950.46 B 1991 and AOAC 952.08 1961 [50,51]. The concentration of carbohydrates was calculated by subtracting the total concentration of the other nutrients.

The DF content of BFB and FeB was assessed in our laboratory following the AOAC Method 2022.01 as reported by Sahin et al. (2023) [37,38].

##### Colour

The colour of the crust and crumb was evaluated using a hand-held colorimeter (Minolta CR-331, Konica Minolta Holdings Inc., Osaka, Japan). Twenty measurements were taken from the crust and twenty-five from the crumb. The evaluations were collected with the CIELAB colour space, where the values L*, a* and b* represent lightness, red/green and yellow/blue values, respectively. To estimate the changes in the colour of the crust and crumb between BFB and FeB, the differential colour index (ΔE) was calculated according to the following equation:

$$\Delta E=\sqrt{{\left(\Delta L^{*}\right)}^{2}+{\left(\Delta a^{*}\right)}^{2}+{\left(\Delta b^{*}\right)}^{2}}$$

where $\Delta L^{*}=L_{BFB}^{*}-L_{FeB}^{*},\;\Delta a^{*}=a_{BFB}^{*}-a_{FeB}^{*},\;\Delta b^{*}=b_{BFB}^{*}-b_{FeB}^{*}$

##### Microbial Shelf Life

To evaluate the shelf life of the bread, the microbial growth was monitored over 14 days using the mould environmental challenge method reported by Sahin, Axel and Arendt (2017) and Dal Bello et al. (2007) with minor amendments [52,53]. Ten centre slices were placed on a sterile metal rack, and both sides of the bread slice were exposed to the environment for 5 min. Each slice was packed in a sterile bag and heat-sealed, and a filter pipette was placed in each bag to allow constant aerobic conditions to predominate. A pre-sterilised and temperature- and humidity-controlled chamber (KOMA SunRiser, Roermond, The Netherlands) was used to store the samples (20 ± 1 °C and 50% RH). The samples were than analysed daily for 14 days in total. The mould growth progression on each bread slice was visually assessed and rated as “mould free”, “mould growth < 10%”, “10–24% mould growth”, “25–49% mould growth” and “mould growth > 50%”.

##### Changes in Texture Property during Storage—Staling

To assess the product’s changes over time, staling was evaluated by measuring crumb hardness using the Texture Profile Analyser as outlined above, spanning five days, as described by Sahin et al. (2018) [54]. The following equation was applied:

$$\mathrm{Staling Rate}=\frac{\mathrm{Crumb}\mathrm{Hardness}\mathrm{at}\;120\;h\;\left[N\right]-\mathrm{Crumb}\mathrm{Hardness}\mathrm{at}\;2\;h\;\left[N\right]}{\mathrm{Crumb}\mathrm{Hardness}\mathrm{at}\;2\;h\;\left[N\right]}$$

##### Bread Microstructure

The bread microstructure was observed using a JEOL Scanning Electron Microscope (JSM-5510, Jeol Ltd., Tokyo, Japan). Prior to sample preparation, the bread crumb was freeze-dried using (LyoQuest, Azbil Telstar, SLU, Barcelona, Spain) for 2 days at −85 °C. A 1.5 mm section of the crumb was cut with a surgical scalpel and positioned on stubs (G 306; 10 mm × 10 mm Diameter; Agar Scientific, Stansted, UK) fixed with carbon tape (G3357N; Carbon Tabs 9 mm; Agar Scientific, Stansted, UK). The samples were then sputter-coated (Polaron E5150 sputter-coating unit) with a gold–palladium blend (ratio of 80:20), and images were captured. The settings for investigation were 5 kV 185 voltage, 20 mm working distance and a magnification factor of 1000.

#### 2.6.3. Release of Reducing Sugar during In Vitro Starch Digestion

An in vitro digestion assay tailored for fibre-enriched products was performed following the methodology outlined by Brennan and Tudorica (2008) [55]. The assay involved enzymatic degradation of starch into reducing sugars over a specified duration. Briefly, 4 g of fresh, crushed bread was exposed to a 30 min proteolytic treatment using pepsin solution. After this, samples were placed in 1-inch-width dialysis tubing, suspended in sodium potassium phosphate buffer (pH 6.9), and incubated for 5 h with a pancreatic α-amylase solution. Samples were taken every 30 min and dialysis tubes were inverted three times every 15 min. To establish the concentration of reducing sugars (maltose) released over time, 100 μL of each sample was diluted with 100 μL of 40 mM 3,5-dinitrosalicyclic acid solution, heated to 100 °C for 15 min and then diluted with 1 mL of deionised water. Analysis was completed in duplicate. Reducing Sugar Release% (RSR%) over time was calculated according to Brennan and Tudorica (2008) using the following formula [55]:

$$\mathrm{RSR}\%=\frac{A_{\mathrm{sample}}\times 500\times 0.95}{A_{\mathrm{maltose}}\times \mathrm{available}\;\mathrm{carbohydrate}}\times 100$$

where A_sample_ is the active sample absorbance at 546 nm, 500 (mL) is the solution total volume, 0.95 is the conversion factor from maltose to starch, A_maltose_ refers to the amount of absorbance for 1 mg of pure maltose in 1 mL of buffer solution, and available carbohydrate represents mg of sugar and digestible starch in 4 g of the sample (values were determined using the Megazyme kit K-RAPRS (Bray, Ireland)). Starch digestibility is presented as reducing sugars released over time. Curve slopes were calculated in Microsoft Excel v. 16.86, ensuring linearity (r^2^ > 0.99).

#### 2.6.4. Statistical Analyses

The measurements were completed in triplicate. Statistical analysis was carried out with GraphPad Prism version 10 (GRAPH PAD Software Inc., Solana Beach, CA, USA); the significance level was set at α≲ 0.05. A *t*-test was used to assess and identify statistically significant differences among the bread and dough characteristics. Various models for the corresponding response factor were assessed using both linear and 2-factorial-interaction in Design Expert to explore fibre–fibre interactions. The significance of each model, as well as each factor/interaction, was analysed. The model needs to be statistically significant, and the lack of fit must be insignificant to determine the inclusion levels of fibre ingredients capable of coexisting in the fibre-enriched bread system. Additionally, a Pearson correlation analysis was carried out to establish correlations between the techno-functional data related to dough and bread data collected.

## 3. Results

### 3.1. Influence of Fibre Ingredients and Fibre-Fibre Interaction in Bread Matrix

**Figure 1:**
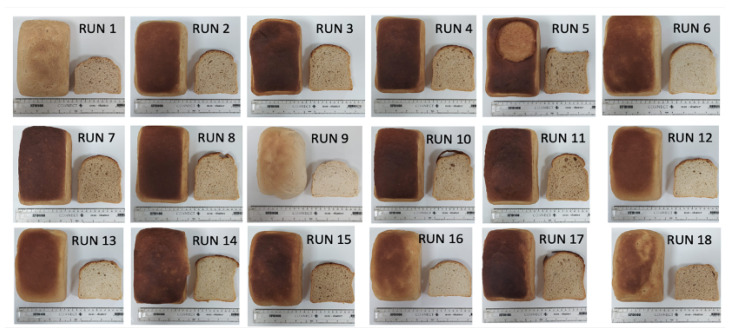
Appearance of the different bread formulations (specific formulation of each run reported in Table 1).

The impact of the addition of soluble (AgriFiber SFC) and insoluble (Fibersym^®^ RW and VITACEL L 600-30) fibre ingredients on bread quality was investigated. Response Surface Methodology was used to investigate the impact of the single fibre ingredients (run 6, run 13, run 16) and combinations. The images of the various bread formulations are shown in Figure 1 and the results are displayed in Table 4.

The inclusion of fibre ingredients in bread changes the overall water absorption. Run 14, not enriched with any fibre ingredients, required a water content of 64.4% to reach a target torque of 500 FU. The replacement of flour with 40% Fibersym^®^ RW (run 6) had the lowest water content (60.5%). In contrast, the addition of AgriFiber SFC or VITACEL L 600-30, both, increased the water addition required to 65.5% and 67%, respectively. The highest water absorption (72.6%) was observed with the combination of 5% AgriFiber SFC and 20% VITACEL L 600-30 (run 15). In contrast, when 5% AgriFiber SFC was combined with 40% Fibersym^®^ RW the water addition was only 65.1%, a significant reduction. The highest fibre addition, 5% AgriFiber SFC, 40% Fibersym^®^ RW and 20% VITACEL L 600-30 (run 18), led to the second highest water addition observed (71.8%). The target torque of 500 BU was not achieved for runs 9 and 12 (442 and 526 BU, respectively), so the level of water addition was adjusted to obtain a dough that was not too dry or sticky and comparable to the control sample.

Bake loss represents the amount of water lost during the baking process. The control bread (run 14) showed a bake loss of 15.94 ± 0.58%, whereas the bake loss of all runs including fibre ingredients ranged between 11.55% and 14.98%. The VITACEL L 600-30 used as a single ingredient resulted in the fibre ingredient that had a greater impact on the bake loss, 13.62% for run 16. Combining 5% AgriFiber SFC with 40% Fibersym^®^ RW or 20% VITACEL L 600-30 led to bake losses of 14.28% and 13.8%, respectively. The combination of 40% Fibersym^®^ RW and20% VITACEL L 600-30 resulted in the lowest bake loss of 11.55%. However, when 5% AgriFiber SFC was added to the previous mixture (run 18), the bake loss increased to 14.68%.

The specific volume is an essential parameter for bread, providing an overview of its rise and expansion after baking. The control bread (run 14) had a specific volume of 4.91 ± 0.09 mL/g, while the fibre-enriched breads showed specific volumes between 2.35 ± 0.01 and 5.04 ± 0.17 mL/g. In general, fibre enrichment tended to decrease the specific volume, except for AgriFiber SFC, which demonstrated the ability to increase the specific volume. The use of the AgriFiber SFC alone, run 13, led to a specific volume of 5.04 ± 0.17 mL/g. When 5% AgriFiber SFC was combined with 40% Fibersym^®^ RW or 20% VITACEL L 600-30, the specific volume decreased to 4.62 mL/g and 3.70 mL/g, respectively. On the other hand, combining 40% Fibersym^®^ RW with 20% VITACEL L 600-30 led to the lowest specific volume of 2.35 mL/g. However, when 5% of AgriFiber SFC was added to the previous mixture (run 18) the specific volume increased to 3.45 mL/g.

The crumb structure was investigated to provide an overview of the slice area (mm^2^), the number of cells and the cell diameter (mm). The control bread (run 14) showed a slice area of 11,440 ± 184 mm^2^, while the slice areas of the fibre-enriched bread runs ranged from between 7107 ± 382 and 12,042 ± 374 mm^2^. Again, the fibre-enriched breads showed a lower slice area except for run 13, which had the maximum inclusion of AgriFiber SFC. The lowest slice area was reported for run 9, which was enriched with the maximum level of Fibersym^®^ RW and VITACEL L 600-30 (7106 mm^2^). However, when 5% AgriFiber SFC was combined with 40% Fibersym^®^ RW or 20% VITACEL L 600-30, slice areas of 10,867 and 9208 mm^2^ were determined, respectively.

The number of cells was increased in all the fibre-enriched runs, with values ranging between 4361 ± 209 and 5563 ± 131. Run 14, without fibre enrichment, showed a cell number of 4325 ± 99.

In line with the other result, the fibre inclusion decreased the cell diameter. A value of 3.01 ± 0.12 mm was reported for run 14, while the fibre-enriched runs showed values ranging from 1.85 ± 0.12 to 2.8 ± 0.08 mm with the exception of run 13 (5% AgriFiber SFC, 3.13 ± 0.19 mm).

The crumb hardness (N) and resilience are characteristics which provide information about the quality of the bread crumb. The control (run 14) had the softest crumb (1.63 ± 0.23 N). The addition of 5% AgriFiber SFC showed the closest value to the control (2.01 ± 0.39 N). In comparison, the inclusion of 40% of Fibersym^®^ RW (run 6) or 20% of VITACEL L 600-30 (run 16) increased the hardness value to 8.04 ± 0.74 and 11 ± 1.06, respectively. The addition of 5% AgriFiber SFC in combination with 40% Fibersym^®^ RW or 20% VITACEL L 600-30 decreased the hardness compared to the runs 6 and 16. The most noteworthy increase was reported for the formulation with 40% of Fibersym^®^ RW and 20% of VITACEL L 600-30 (run 9), resulting in a crumb hardness of 32.46 ± 2.44 N.

Furthermore, the control (run 14) showed higher crumb resilience (0.59 ± 0.02), while fibre fortification led to values between 0.24 and 0.51. The addition of 5% AgriFiber SFC showed a close value to the control (0.51 ± 0.02). The combination of 40% Fibersym^®^ RW and 20% VITACEL L 600-30 (run 9) showed the second lowest crumb resilience (0.28 ± 0.01). The addition of 5% AgriFiber SFC to this mixture (run 18) further decreased crumb resilience (0.24 ± 0.03).

The water activity was determined to investigate the bread matrix’s available water. The values for all breads ranged between 0.94 and 0.98. Hence, the addition of the fibre ingredients did not influence the water activity considerably.

### 3.2. Combination of Fibre Ingredients in Dough and Bread Matrix

**Figure 2:**
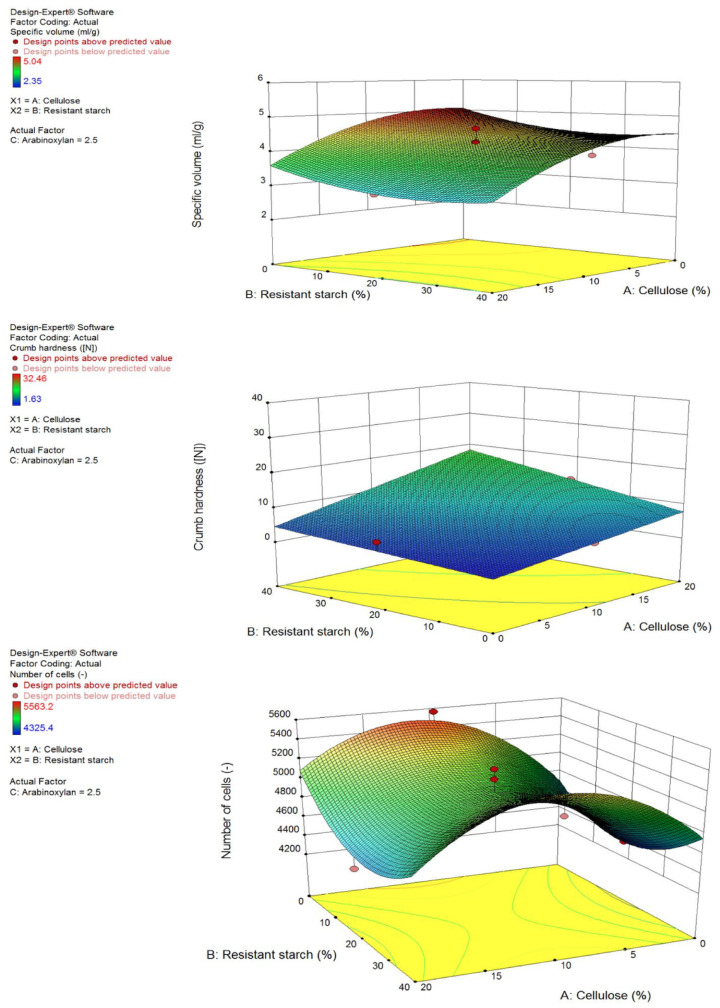
Three-dimensional surface plots of the results of the response surface method (RSM) for specific volume, crumb hardness and numbers of cells based on a constant concentration of 2.5% AgriFiber SFC plus variations of Fibersym RW and VITACEL L 600-30.

To identify the optimal combination and concentration of DF ingredients that can coexist in the bread model system with minimal impact on bread properties, the proposed model by Design Expert must be statistically significant and show an insignificant lack-of-fit. The statistical significance was analysed for each parameter (Table A1). The significance of fiber-fiber interactions across different runs was assessed through statistical evaluation. Additionally, the models have been reported as 3D-surface plots (Figure 2). The inclusion of AgriFiber SFC increased the specific volume, however, Fibersym^®^ RW and VITACEL L 600-30 decreased it. Overall, the specific volume model was found to be significant with an insignificant lack-of-fit. The same results of model and lack-of-fit were found in the numbers of cells. All the fibre ingredients inclusion were found to be significant for the model but significant also for the lack-of-fit. Based on the data collected and the statistical analyses, the Design Expert optimisation tool determined the optimal fibre-enriched bread formulation (FeB) in which 53% of flour was replaced with 40% Fibersym^®^ RW, 11% VITACEL L 600-30, and 2% AgriFiber SFC.

#### 3.2.1. Dough Analysis

Dough analyses were conducted to investigate the gluten network formation and dough development, as well as the starch pasting properties, yeast performance, and viscoelastic properties (Table 5).

**Figure 3:**
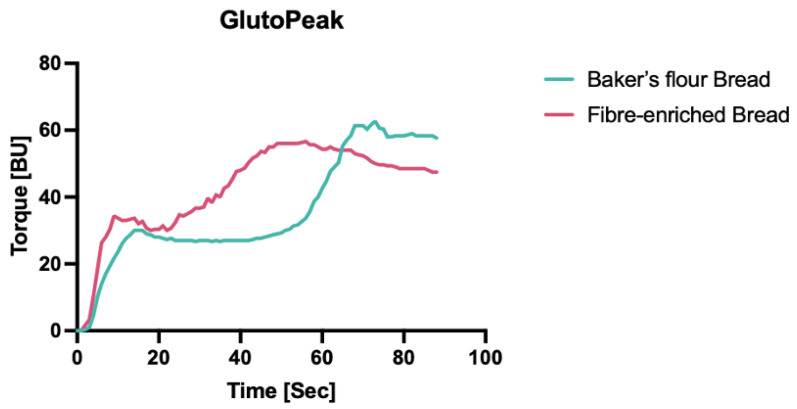
GlutoPeak profiles of baker’s flour control and fibre-enriched mixture. Gluten network development characterisation, as dough torque (BU) over mixing time (s).

The difference in gluten aggregation was investigated characterising gluten network strength and developing time. Figure 3 illustrates the torque (BU) plotted against the time (s) during the development of the gluten network. The BFB showed a TM of 64.67 ± 1.15 BU and a PMT of 71.00 ± 2.65 s, while the FeB had a gluten network strength of 56.67 ± 0.58 BU and a development time of 53.67 ± 2.52 s. Hence, fibre inclusion significantly reduced the gluten network strength and the time required for its development.

The Mixolab characterised the rheological behaviour and pasting properties of the different doughs. BFB and FeB did not show a significant difference in the dough development time (DDT), with values of 1.09 ± 0.13 min and 0.87 ± 0.12 min determined, respectively. The highest C2 value was observed for the BFB (0.44 ± 0.01 Nm), which differed significantly from FeB (0.39 ± 0.01 Nm). Similar trends were observed for other parameters, including C4, and C5. However, the C3 value was higher for FeB (2.22 ± 0.08 Nm) compared to the BFB (1.71 ± 0.04 Nm).

The yeast fermentation capability was investigated to compare how the bread matrix would behave during the fermentation process. The BFB had the highest Hm (58.30 ± 2.26 mm), while the Hm of FeB (35.80 ± 3.70 mm) was reduced by 38.59%. The CO_2_ production during the fermentation process was 2047 ± 26 mL for BFB and 1587 ± 20 mL for FeB. However, the CO_2_ retention coefficient did not significantly differ between BFB (99.50 ± 0.17%) and FeB (99.63 ± 0.06%).

Oscillatory measurements were performed to study the dough’s elastic and viscous behaviour. The BFB showed a damping factor of 0.33 ± 0.00, which was significantly higher compared to FeB (0.30 ± 0.01). Hence, fibre addition, resulted in a higher elastic behaviour.

#### 3.2.2. Bread Quality

**Figure 4:**
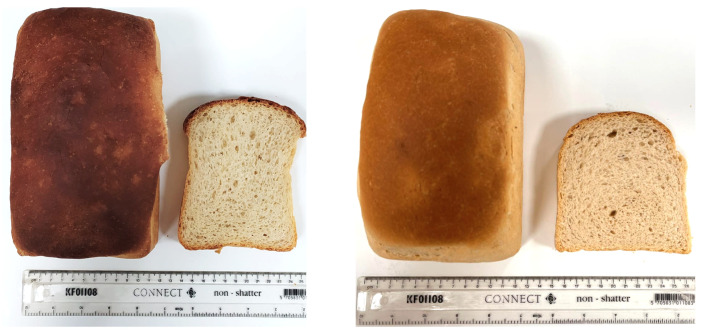
Images of the Baker’s flour control bread (BFB, (**left**)) and Fibre-enriched bread (FeB, (**right**)). A loaf and a central slice have been captured for each bread.

Besides investigating the impact of DF ingredients on bread quality, the aim of this study was to optimise the fibre fortification level to resemble a white wheat bread rich in fibre. A visual representation of the BFB and FeB can be found in the Figure 4 and an overview of the result can be found in the Table 6.

The results of the compositional analyses are shown in Table 7. The fibre analyses revealed a two-fold higher concentration of DF in FeB compared to the BFB; the FeB contained 9.15 ± 1.06 g of DF. Correspondingly, the partial replacement of flour in the FeB significantly decreased the total sugar content of the breads (BFB 2.05 ± 0.14; FeB 0.92 ± 0.07 g/100 g). Levels of the other macronutrients were comparable in BFB and FeB.

The fibre fortification caused a significant decrease in bake loss, 13.53% in FeB compared to 14.53% in BFB. Furthermore, the specific volume decreased significantly by 16.4% (BFB 4.94 ± 0.21; FeB 4.13 ± 0.18).

The crumb structure analyses revealed that the slice area and cell diameter for both BFB and FeB align with the findings from the RSM runs. The slice area and cell diameter of the BFB were 11,868 ± 378 mm^2^ and 2.38 ± 0.13 mm, respectively, while the FeB values were 9964 ± 470 mm^2^ and 2.29 ± 0.15 mm. In contrast, the number of cells is lower in the FeB (5728 ± 363) compared to the BFB (5309 ± 352), decreasing by 7.3%.

The fibre fortification significantly increased crumb hardness from 2.14 ± 0.24 N in BFB to 8.92 ± 1.59 N in FeB. Also, there was a significant difference in the crumb resilience between BFB (0.56 ± 0.02 N) and the FeB (0.29 ± 0.02 N).

Comparing BFB and FeB, comparable water activity values were reported for both products (0.97 ± 0.01).

Variations in the crust and crumb colour of the breads were assessed through δE values compared to BFB, considering differences in the L*, a*, and b* colour values. A significant difference was observed in the FeB in both crust (17.13 ± 7.57) and crumb (6.63 ± 2.45).

**Figure 5:**
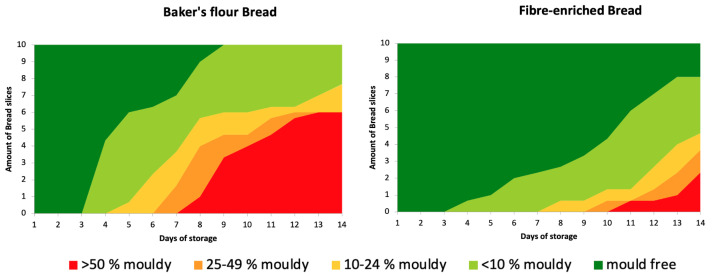
Microbial shelf life evaluation of Baker’s flour control bread (BFB) and Fibre-enriched bread (FeB) over 14 days.

The results of the microbial shelf-life are displayed in the Figure 5. In both BFB and FeB, the first mould (less than 10% covered) appeared after three days. However, the kinetics of mould growth showed a slower growth in FeB compared to the BFB. The second mould stage (10–24% mouldy) started after four days in BFB and after seven days in FeB, significantly delayed the overall growth.

BFB showed the highest rate of staling (3.18 ± 0.75) whereas a significant decrease in the staling rate was observed in FeB (0.99 ± 0.40).

**Figure 6:**
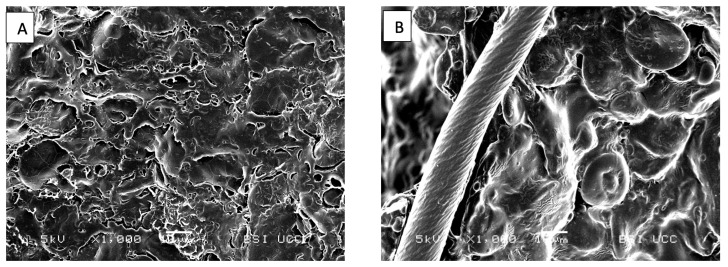
SEM micrographs of freeze-dried crumb on day of baking. Pictures illustrate Baker’s flour control bread (BFB, (**A**)) and Fibre-enriched bread (FeB, (**B**)).

SEM was used to investigate the crumb microstructure (Figure 6). Figure 6A presents partially gelatinised, porous starch granules embedded in a protein matrix for the BFB. Compared with the Figure 6B, a higher level of intact, defined, and exposed starch granules can be seen. In addition, the starch granules are covered by a film-like matrix and a cellulose filament is visible on the left front side of the image.

#### 3.2.3. Impact of Fiber on Starch Digestibility

**Figure 7:**
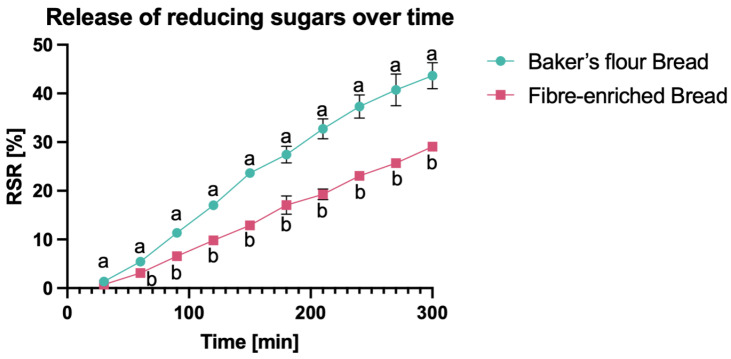
Release of reducing sugars during in-vitro starch hydrolysis/digestion of fresh bread samples over 300 min. The data are presented as mean ± standard deviation. Points of the curve marked by different letters in the same line are significantly different (*p* < 0.05).

Starch digestibility was assessed using an in-vitro model system, examining the release of reducing sugar (RSR) during a simulated starch digestion process. The results are showed in Figure 7. The RSR over time was the highest in BFB, indicated by the slope 0.15 maltose released (%)/min. In comparison, FeB showed a lower RSR over time with a slope of 0.10 maltose released (%)/min. Adding fibre reduced the bread starch digestibility, leading to a decreased release of sugars.

## 4. Discussion

Increasing DF in the Western diet is essential to meet nutritional requirements [23,56,57]. However, population-wide fibre consumption remains low, most consumers prefer refined products over high-fibre wholesome foods, such as whole grain [10]. Fortifying staple foods and widely consumed products with purified fibre ingredients could facilitate an elevated DF intake. Nevertheless, current research largely focuses on the application of single fibres, emphasising the requirement for more investigation into combinations of fibres.

This study offers a comprehensive examination of the interactions among resistant starch (Fibersym^®^ RW), a purified insoluble fibre ingredient (VITACEL L 600-30), and a soluble fibre (AgriFiber SFC) in a bread matrix. Moreover, the application of an optimisation tool resulted in a mixture in which the fibre ingredients could coexist without impacting bread quality in a major way. DF has been reported to compete with macronutrients (protein, starch and sugar) for water within different food matrices [58,59].

The inclusion of AgriFiber SFC, a soluble fibre, resulted in breads with a higher specific volume and lower bake loss, which could be attributed to improved water retention [21,60]. However, this ingredient had the most significant impact on the colour of the products due to the brown/gold shade of the raw material, resulting in a final maximum addition level of 2%. The addition of insoluble fibres, Fibersym^®^ RW and VITACEL L 600-30, resulted in a lower specific volume, which may be attributed to their physical interaction with the gluten strands leading to a weakening of the network and hence a lower rise during proofing [34,35,61,62]. Overall, the inclusion of the individual fibre ingredients and their different combinations resulted in increased crumb hardness and lower resilience. As previously mentioned, Fibersym^®^ RW and VITACEL L 600-30 decreased the bread volume, thus resulting in a denser crumb and increased crumb hardness; on the other hand, AgriFiber SFC increased crumb hardness due to its recrystallisation effect after baking [34,35,60,61,62].

In addition to evaluating the performance of individual fibre ingredients added to the bread matrix, the experimental design offered an insight into fibre–fibre interactions. Comparing the impact of the combination of soluble–insoluble fibre ingredients and insoluble–insoluble fibre ingredients on the bread quality found that the latter had a more detrimental effect on the final product. The mixture of Fibersym^®^ RW and VITACEL L 600-30 resulted in the greatest negative impact on bake loss, specific volume, and especially hardness. Despite these results, the inclusion of AgriFiber SFC alongside these fibre ingredients led to an improvement in bread characteristics, which were more comparable to the control (BFB). A study conducted by Zhao et al. (2020) reported that the incorporation of a water-extractable arabinoxylan, such as AgriFiber SFC, weakened the viscoelasticity of non-heated gluten while further enhancing the viscoelasticity during heating [63]. These findings suggest that the incorporation of AgriFiber SFC positively influences both the rheological properties of dough and the quality of bread products.

The composition of both BFB and FeB bread aligns with the industry’s standards for white bread production [64,65]. Although, as a result of the inclusion of the optimal combination of AgriFiber SFC, Fibersym^®^ RW and VITACEL L 600-30, FeB resulted in high-fibre bread, in accordance with the EU regulation [66], thus improving nutritional value. A single serving size of the control bread, typically around 50 g in Europe, provides 2 g of DF, covering approximately 7% of the recommended daily intake (around 30 g of DF). In contrast, consuming the same amount of the optimised bread yields 4.6 g of fibre, covering 15% of the recommended intake. It appears that these ingredients had an impact on both the dough and the overall quality of the bread, by interacting with the gluten strands and impacting the recrystallisation during the cooling process, as previously explained.

The strength of the gluten network and the duration required for its development are essential factors to ensure both optimal dough consistency and high-quality bread. A lower torque max and a shorter peak max time were reported for the FeB dough compared to the BFB dough, resulting in a weaker network and a faster development time. Incorporating fibre ingredients accelerates the kinetics of the gluten network aggregation, interferes with the secondary structure of gluten proteins, and limits the hydration of the gluten network [63,67,68].

Additionally, a comprehensive examination of the proteins and starch pasting behaviour during mixing and heating by Mixolab analysis offers an additional understanding of bread quality. The decrease in the DDT is in line with the lower value of PMT, which supports the theory of an accelerated kinetic aggregation. The decrease in C2 values suggests a weakening effect on protein due to the inclusion of fibre ingredients, supporting the previously observed results of reduced gluten network strength. The increase in C3 value suggested a higher level of starch gelatinisation, in contrast with what is usually reported in fibre-enrichment research [69,70,71]. This is likely linked to the presence of AgriFiber SFC due to its gelling properties [72]. C5, which represents the starch retrogradation, was lower in the FeB. This result is in line with the findings of the GlutoPeak analyses. As a result of fibre mixture addition, the dough may not develop sufficient structure during mixing, leading to weaker dough and lower C5 values. This value is known to be negatively correlated with the shelf life [73]. Additionally, the lower starch retrogradation can be linked to the lower staling rate in the FeB. The inclusion of fibre ingredients is known to halve water-binding capacity and prevent starch recrystallisation, which have an impact on the staling rate [62,74,75]. The lower staling rate also supports the theory of a longer shelf life.

The evaluation of bread fermentation revealed a decrease in the total volume of CO_2_ produced as a result of the inclusion of fibre ingredients. Replacing part of the BF with the fibre mixture decreased the available substrate for yeast fermentation [76], leading to a decrease in the maximum height of gaseous release (Hm).

Additionally, the decrease in the damping factor indicated an increase in the dough’s elastic portion. Multiple studies have shown that the insoluble fibres tend to increase the elastic module [77,78], due to their mechanical hindrance. The partial substitution of flour with the optimal mixture led to a firmer dough that exhibited increased resistance to deformation, as observed by Neylon et al. (2021) [42]. Notably, no significant correlation was discerned between the damping factor and Hm (r = 0.22). Consequently, the rise of the dough was compromised due to the substitution of digestible carbohydrates and relative halving of the sugar concentration in FeB.

Gluten network and dough development time had a significant positive correlation with specific volume (r = 0.91, *p* < 0.01 and r = 0.82, *p* < 0.05, respectively). The restriction imposed on gluten network quality and the lower volume of CO_2_ produced during the fermentation led to a lower specific volume and lower slice area. The variations observed in crumb hardness may be attributed to the differences noted in the dough damping factor and the bread specific volume, as crumb hardness had a strong negative correlation with these two parameters (r = —0.85, *p* < 0.01, r = —0.93, *p* < 0.01, respectively). Analysis of the crust and crumb colour revealed differences in both attributes. The variance in crumb colour can be attributed to the inclusion of AgriFiber SFC, as reported in the previous paragraph. Furthermore, the disparity in crust colour may be linked to the mixture of fibre ingredients. The browning of the crust was a result of the Maillard reaction, which occurs between reducing sugars and proteins [79,80]. The reduction in the sugar concentration, as highlighted in the nutritional profile of FeB, resulted in a lower degree of the Maillard reaction and, consequently, a colour difference.

Observations of microbial shelf-life kinetics revealed the presence of mould after three days in both BFB and FeB. This result was in line with the water activity values of the two breads, which did not significantly differ. However, the subsequent growth of mould was significantly slower in FeB, potentially attributed to the partial replacement of BF and decreased availability of substrate (easily digested carbohydrates) for microbial growth. A denser crumb structure may also be responsible for impeding the aeration needed for microbial growth [42,81].

The addition of fibre ingredients resulted in structural changes in the bread matrix. This, in turn, led to a reduction in sugar release during starch digestion in FeB. As previously mentioned, the ultrastructure of FeB showed embedded starch granules covered by a film-like matrix, which reduced the accessibility of starch to amylase. Most of the starch granules are associated with the incorporation of Fibersym^®^ RW. The inclusion of ingredients such as Fibersym^®^ RW has been reported to significantly lower the RSR of bread, putatively, due to its modified structure by phosphorylated cross-linked starch, which hinders the binding of amylase to starch [38,82,83]. Additionally, during the cooling process, starch molecules re-organise to form a closely packed structure using hydrogen bonding. This results in lower enzyme activity on the substrate [84,85], which is further slowed down by the protective layer created by AgriFiber SFC surrounding the starch granules. Additionally, intact cellulose filaments, which are resistant to digestion [86], might further slow digestion.

## 5. Conclusions

Incorporating purified blends of fibre ingredients into various food products has been challenging. Nevertheless, this study marks a promising initial step towards incorporating a mixture of DF into bread, mimicking the product quality of white wheat bread. A mixture of fibre ingredients, varying in source, structure and characteristics such as solubility, fermentability and functionality, could enhance human health and improve food quality. This study highlights the impact of three types of DF ingredients on bread quality and nutrition, and their interactions with each other, resulting in a bread in which they can coexist. Combining these three DF ingredients in the optimal concentration achieved a high-fibre bread with a superior nutritional profile compared to white flour bread, successfully closing the fibre gap. The fibre inclusion in the FeB significantly decreased the release of reducing sugar in vitro, as compared to the control product. Additionally, the proliferation of microbial populations on fibre-enriched products exhibited a decelerated growth rate. This represents a crucial contribution to the existing body of research, providing valuable insights into how this mixture interplay influences the overall quality and nutritional value of optimised high-fibre bread.

## Figures and Tables

**Table 1:** Fibre and protein concentration of baker’s flour (BF,) dietary fibre ingredients and vital gluten on an “as is” basis. Data are presented as mean ± standard deviations.

	Baker’s Flour	AgriFiber SFC	Fibersym^®^ RW	VITACEL L 600-30	Vital Gluten
g fibre/100 g	7.74 ± 0.23	45.96 ± 0.43	47.86 ± 0.89	96.62 ± 1.22	−
g protein/100 g	12.96 ± 0.79	5.48 ± 0.5	0.07 ± 0.01	0.08 ± 0.02	76.74 ± 1.47

**Table 2:** Experimental design for the investigation of fibre–fibre interactions in a bread system reporting the baker’s flour, different fibre ingredients and vital gluten concentration. The concentration of the ingredients is based on baker’s flour and fibre ingredients as 100% total [%]. FeB (fibre-enriched bread) represents the final combination of the fibre ingredients based on the results of the single runs and in situ optimisation by Response Surface Methodology.

Run	Baker’s Flour [%]	AgriFiber SFC [%]	Fibersym^®^ RW [%]	VITACEL L 600-30 [%]	Vital Gluten [%]
1	57.5	2.5	20	20	6.96
2	47.5	2.5	40	10	8.65
3	67.5	2.5	20	10	5.28
4	67.5	2.5	20	10	5.28
5	77.5	2.5	20	0	3.60
6	60	0	40	0	6.72
7	65	5	20	10	5.53
8	67.5	2.5	20	10	5.28
9	40	0	40	20	10.08
10	67.5	2.5	20	10	5.28
11	55	5	40	0	7.21
12	70	0	20	10	5.04
13	95	5	0	0	0.49
14	100	0	0	0	-
15	75	5	0	20	3.85
16	80	0	0	20	3.36
17	87.5	2.5	0	10	1.92
18	35	5	40	20	10.57
FeB	47	2	40	11	8.76

**Table 3:** Baker’s flour control bread recipe expressed as percentages based on flour [%].

Ingredients	Amount [%]
Baker’s flour	100
Sunflower oil	3.2
Salt	1.2
Sugar	2
Yeast	2
Water	64.4

**Table 4:** Characteristics of breads fortified with different amounts and combinations of VITACEL L 600-30, Fibersym^®^ RW and AgriFiber SFC.

Run	AgriFiber SFC [%]	Fibersym^®^ RW [%]	VITACEL L 600-30 [%]	Vital Gluten [%]	Water Addition [%]	Bake Loss [%]	Specific Volume [mL/g]	Slice Area [mm^2^]	Number of Cells	Number of Holes	Area of Cells [%]	Cell Diameter [mm]	Crumb Hardness [N]	Crumb Resilience [−]	Water Activity [−]
1	20	20	2.5	6.96	68.1	14.98 ± 0.27	2.99 ± 0.02	7498 ± 354	4424 ± 301	1.64 ± 0.79	50.52 ± 0.34	2.01 ± 0.11	13.06 ± 1.52	0.3 ± 0.03	0.94 ± 0.02
2	10	40	2.5	8.65	66	14.12 ± 0.26	4.04 ± 0.04	10,015 ± 395	5127 ± 356	1.51 ± 0.91	53.54 ± 0.83	2.33 ± 0.16	7.93 ± 0.93	0.3 ± 0.02	0.95 ± 0
3	10	20	2.5	5.28	66	13.7 ± 0.41	4.24 ± 0.05	10,594 ± 277	5019 ± 226	1.27 ± 1.11	54.8 ± 0.91	2.5 ± 0.15	6.06 ± 0.63	0.38 ± 0.02	0.97 ± 0.01
4	10	20	2.5	5.28	66	14.6 ± 0.77	4.25 ± 0.08	10,372 ± 215	4909 ± 189	1.01 ± 0.62	54.66 ± 0.73	2.5 ± 0.1	6.51 ± 0.71	0.39 ± 0.01	0.97 ± 0
5	0	20	2.5	3.60	63.4	14.48 ± 0.65	4.18 ± 0.21	9938 ± 308	4361 ± 209	0.84 ± 0.67	55.65 ± 0.43	2.8 ± 0.08	5.38 ± 1.2	0.43 ± 0.01	0.96 ± 0.02
6	0	40	0	6.72	60.5	14.49 ± 0.3	4.23 ± 0.03	9121 ± 2684	4596 ± 1326	1.24 ± 0.85	49.91 ± 13.87	2.22 ± 0.65	8.04 ± 0.74	0.4 ± 0.01	0.94 ± 0.01
7	10	20	5	5.53	67	14.98 ± 0.31	4.34 ± 0.23	9623 ± 2518	4726 ± 1293	1.15 ± 0.85	50.67 ± 13.35	2.28 ± 0.62	5.47 ± 0.67	0.34 ± 0.01	0.97 ± 0.01
8	10	20	2.5	5.28	66	14.3 ± 0.67	4.21 ± 0.1	10,241 ± 176	5158 ± 182	0.54 ± 0.62	53.63 ± 0.24	2.3 ± 0.09	6.06 ± 0.75	0.4 ± 0.03	0.97 ± 0
9	20	40	0	10.08	65	11.55 ± 0.16	2.35 ± 0.01	7106 ± 381	4735 ± 280	1.96 ± 1.35	49.28 ± 0.5	1.92 ± 0.12	32.46 ± 2.44	0.28 ± 0.01	0.98 ± 0.01
10	10	20	2.5	5.28	66	14.43 ± 0.03	4.65 ± 0.13	11,274 ± 349	5258 ± 166	0.96 ± 1.16	54.68 ± 0.64	2.53 ± 0.13	4.1 ± 0.82	0.41 ± 0.02	0.95 ± 0
11	0	40	5	7.21	65.1	14.28 ± 0.21	4.62 ± 0.01	10,867 ± 486	4736 ± 124	0.49 ± 0.49	55.91 ± 0.82	2.73 ± 0.16	4.04 ± 0.56	0.33 ± 0.02	0.95 ± 0.02
12	10	20	0	5.04	60.8	13.52 ± 0.21	3.48 ± 0.01	8956 ± 279	4973 ± 139	2.12 ± 0.91	52.4 ± 0.33	2.29 ± 0.11	10.32 ± 1.18	0.45 ± 0.01	0.97 ± 0.01
13	0	0	5	0.49	65.6	14.87 ± 0.36	5.04 ± 0.17	12,042 ± 373	4761 ± 133	0.36 ± 0.46	57.77 ± 0.6	3.13 ± 0.19	2.01 ± 0.39	0.51 ± 0.02	0.97 ± 0
14	0	0	0	0	64.4	15.94 ± 0.58	4.91 ± 0.09	11,440 ± 184	4325 ± 99	0.13 ± 0.31	56.43 ± 0.24	3.01 ± 0.12	1.63 ± 0.23	0.59 ± 0.02	0.96 ± 0.01
15	20	0	5	3.85	72.6	13.8 ± 0.56	3.7 ± 0.02	9208 ± 224	4667 ± 207	1.2 ± 1.54	54.14 ± 0.91	2.31 ± 0.14	7.08 ± 0.54	0.41 ± 0.01	0.97 ± 0
16	20	0	0	3.36	67	13.62 ± 0.28	3.19 ± 0.11	8243 ± 359	5249 ± 355	1.14 ± 0.88	50.52 ± 0.88	1.85 ± 0.12	11 ± 1.06	0.45 ± 0.01	0.97 ± 0
17	10	0	2.5	1.92	66.8	13.97 ± 0.5	4.69 ± 0.15	11,564 ± 353	5563 ± 131	1.58 ± 0.93	55.35 ± 0.71	2.5 ± 0.1	4.52 ± 0.59	0.47 ± 0.01	0.97 ± 0.01
18	20	40	5	10.57	71.8	14.68 ± 0.36	3.45 ± 0.09	867 ± 261	4642 ± 159	1.58 ± 1.25	52.54 ± 0.38	2.25 ± 0.12	8.72 ± 0.98	0.24 ± 0.03	0.98 ± 0

**Table 5:** Dough characteristics of Baker’s flour control bread (BFB) and Fibre-enriched bread (FeB). The data are presented as mean ± standard deviation. Values followed by different letters in the same column are significantly different (*p* < 0.05).

Sample	Water [%]	Torque Max [BU]	Peak Max Time [s]	DDT [min]	C2 [Nm]	C3 [Nm]	C4 [Nm]	C5 [Nm]	Hm [mm]	Vol. of CO_2_ [mL]	CO_2_ Retention [%]	Damping Factor
BFB	64.4	64.67 ± 1.15 ^a^	71.00 ± 2.65 ^a^	1.09 ± 0.13 ^a^	0.44 ± 0.01 ^a^	1.71 ± 0.04 ^a^	1.59 ± 0.01 ^a^	2.43 ± 0.01 ^a^	58.30 ± 2.26 ^a^	2047 ± 26.16 ^a^	99.50 ± 0.17 ^a^	0.33 ± 0.00 ^a^
FeB	69	56.67 ± 0.58 ^b^	53.67 ± 2.52 ^b^	0.87 ± 0.12 ^a^	0.39 ± 0.01 ^b^	2.22 ± 0.08 ^b^	1.52 ± 0.03 ^b^	1.96 ± 0.06 ^b^	35.80 ± 3.70 ^b^	1587 ± 19.67 ^b^	99.63 ± 0.06 ^a^	0.30 ± 0.01 ^b^

DDT, dough development time.

**Table 6:** Bread characteristics of BF bread and Fibre-enriched bread. The data are presented as mean ± standard deviation. Values followed by different letters in the same column are significantly different (*p* < 0.05).

Product	Bake Loss [%]	Specific Volume [mL/g]	Slice Area [mm^2^]	Number of Cells	Number of Holes	Cell Diameter [mm]	Crumb Hardness [N]	Stale Rate	Crumb Resilience	Water Activity [−]	ΔE Crust	ΔE Crust
BFB	14.53 ± 0.32 ^a^	4.94 ± 0.21 ^a^	11868 ± 378 ^a^	5728 ± 363 ^a^	1.53 ± 1.05 ^a^	2.38 ± 0.13 ^a^	2.14 ± 0.24 ^a^	3.18 ± 0.75 ^a^	0.56 ± 0.02 ^a^	0.97 ± 0.01 ^a^	-	-
FeB	13.39 ± 0.30 ^b^	4.13 ± 0.18 ^b^	9964 ± 470 ^b^	5309 ± 352 ^b^	1.32 ± 0.78 ^b^	2.29 ± 0.15 ^b^	8.92 ± 1.59 ^b^	0.99 ± 0.40 ^b^	0.29 ± 0.02 ^b^	0.97 ± 0.01 ^a^	17.13 ± 7.57	6.63 ± 2.45

**Table 7:** Composition of the Baker’s flour control bread (BFB) and Fibre-enriched bread (FeB), expressed as g/100 g. The values, exception made for the fibre concentration, were gain from an external laboratory. The standard deviation follow their internal standards and was omitted from the table. The fibre values are presented as mean ± standard deviation. The significant difference (*p* < 0.05) is represented by different letters.

	Baker’s Flour Bread	Fibre-Enriched Bread
Energy [kcal/100 g]	266	268
Total fats [g/100 g]	3.05	2.95
Saturated fats [g/100 g]	0.46	0.43
Carbohydrates [g/100 g]	50.89	51.87
Sugar [g/100 g]	2.05	0.92
Fibre [g/100 g]	4.02 ± 1.17 ^a^	9.15 ± 1.06 ^b^
Proteins [g/100 g] (N × 6.25)	8.74	8.47
Moisture [g/100 g]	36.10	35.43
Ash [g/100 g]	1.22	1.28
Sodium [g/100 g]	0.33	0.38

## Data Availability

The original contributions presented in the study are included in the article. Further inquiries can be directed to the corresponding author.

## Appendix A. Response Surface Methodology—ANOVA Analyses

**Table A1:** Statistical analyses, ANOVA, of the models of the response factors.

Response Factor	Model	ANOVA Models			ANOVA Lack-of-Fit		
		**F-Value**	***p*** **-Value**	**Significance**	**F-Value**	***p*** **-Value**	**Significance**
**Specific volume [mL/g]**	Linear	16.02	0.00	S.	1.47	0.40	N.S.
	A	94.24	0.00	S.			
	B	14.26	0.01	S.			
	C	15.81	0.00	S.			
	AB	0.00	0.99	N.S.			
	AC	2.63	0.14	N.S.			
	BC	1.60	0.24	N.S.			
**Bake loss [%]**	Linear	4.52	0.02	S.	2.82	0.21	N.S.
	A	8.29	0.01	S.			
	B	2.66	0.13	N.S.			
	C	3.42	0.09	N.S.			
	AB	0.25	0.62	N.S.			
	AC	7.40	0.02	S.			
	BC	5.10	0.05	S.			
**Crumb hardness [N]**	Linear	13.30	0.00	S.	9.85	0.04	S.
	A	30.45	0.00	S.			
	B	14.18	0.00	S.			
	C	15.15	0.00	S.			
	AB	3.12	0.11	N.S.			
	AC	8.39	0.01	S.			
	BC	8.50	0.01	S.			
**Crumb resilience [N]**	Linear	120.24	0.00	S.	2.32	0.26	N.S.
	A	98.94	0.00	S.			
	B	227.76	0.00	S.			
	C	34.00	0.00	S.			
	AB	-	-	-			
	AC	-	-	-			
	BC	-	-	-			
**Water activity [-]**	Linear	-	-	-	0.06	1.00	N.S.
	A	-	-	-			
	B	-	-	-			
	C	-	-	-			
	AB	-	-	-			
	AC	-	-	-			
	BC	-	-	-			
**Cell diameter [mm]**	Linear	19.60	0.00	S.	1.60	0.38	N.S.
	A	81.11	0.00	S.			
	B	11.71	0.01	S.			
	C	12.69	0.00	S.			
	AB	11.34	0.01	S.			
	AC	0.25	0.63	N.S.			
	BC	0.50	0.49	N.S.			
**Number of cells**	Linear	5.65	0.01	S.	1.64	0.36	N.S.
	A	2.67	0.14	N.S.			
	B	1.61	0.24	N.S.			
	C	0.36	0.57	N.S.			
	AB	2.33	0.17	N.S.			
	AC	5.93	0.04	S.			
	BC	0.14	0.72	N.S.			
**Number of holes**	Linear	6.37	0.01	S.	1.82	0.34	N.S.
	A	13.25	0.00	S.			
	B	3.70	0.08	N.S.			
	C	2.16	0.16	N.S.			
	AB	-	-	-			
	AC	-	-	-			
	BC	-	-	-			
**Area of cells**	Linear	8.59	0.00	S.	10.90	0.04	S.
	A	13.30	0.00	S.			
	B	6.50	0.02	S.			
	C	5.97	0.03	S.			
	AB	-	-	-			
	AC	-	-	-			
	BC	-	-	-			

A, VITACEL L-600-30; B, FiberSym RW; C, AgriFiber SFC; S., significant; N.S., not significant.
